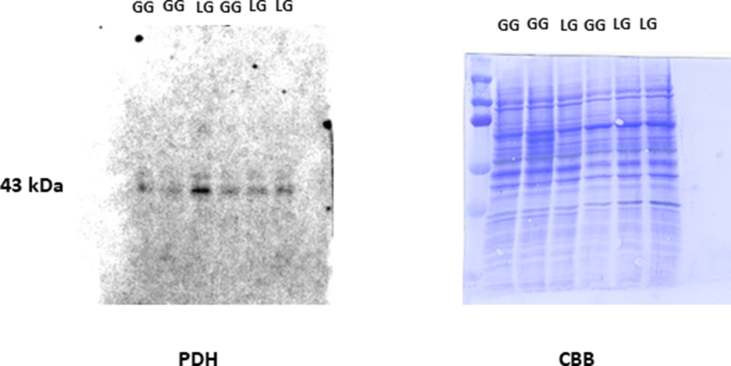# Chronic lactate exposure promotes cardiomyocyte cytoskeleton remodelling

**DOI:** 10.1016/j.heliyon.2024.e24719

**Published:** 2024-01-16

**Authors:** Simone Luti, Rosamaria Militello, Gabriella Pinto, Anna Illiano, Riccardo Marzocchini, Alice Santi, Matteo Becatti, Angela Amoresano, Tania Gamberi, Alessio Pellegrino, Alessandra Modesti, Pietro Amedeo Modesti

**Affiliations:** a Department of Biomedical, Experimental and Clinical Sciences “Mario Serio”, University of Florence, Florence, Italy; b Department of Chemical Sciences, University of Naples Federico II, Naples, Italy; c Department of Experimental and Clinical Medicine, University of Florence, Florence, Italy

**Keywords:** Lactate, Cardiomyocytes, Metabolomic, Proteomic, Adaptation, Cytoskeleton, ROS

## Abstract

We investigated the effect of growing on lactate instead of glucose in human cardiomyocyte assessing their viability, cell cycle activity, oxidative stress and metabolism by a proteomic and metabolomic approach. In previous studies performed on elite players, we found that adaptation to exercise is characterized by a chronic high plasma level of lactate. Lactate is considered not only an energy source but also a signalling molecule and is referred as “lactormone”; heart is one of the major recipients of exogenous lactate. With this in mind, we used a cardiac cell line AC16 to characterize the lactate metabolic profile and investigate the metabolic flexibility of the heart. Interestingly, our data indicated that cardiomyocytes grown on lactate (72 h) show change in several proteins and metabolites linked to cell hypertrophy and cytoskeleton remodelling. The obtained results could help to understand the effect of this metabolite on heart of high-performance athletes.

## Highlights

• Chronic lactate exposure induced an adaptation phase in cardiomyocytes.
• Lactate affect cardiomyocytes metabolism.
• Chronic lactate exposure promotes cardiomyocytes cytoskeletal remodelling.
• Chronic lactate exposure sustain succinate increase.

## 1 Introduction

In several previous studies performed on male and female elite basketball players, we found that adaptation to exercise is characterized by higher antioxidant capacity, reduced oxidative species, increased levels of hormones [[1], [2], [3], [4], [5]]. Our analysis were performed 24 h after training sessions and, interestingly, among other metabolites we found an increased level of plasma lactate in comparison to sedentary subjects, suggesting that there is a general lactate elevation in players [3]. The production of lactate has been widely used as a biomarker to reflect exercise mode, strength, and duration [6].

Although lactate has traditionally been viewed as a metabolic waste product there has been a revolution in understanding its role in normal and pathophysiological conditions [7].

By shuttling between producer (driver) and consumer (recipient) cells lactate fulfils at least three purposes: (i) a major energy source for mitochondrial respiration; (ii) the major gluconeogenic precursor; and (iii) a signalling molecule with autocrine-, paracrine- and endocrine-like effects and is referred to as a “lactormone” [7].

Examples of the cell-cell lactate shuttles include lactate exchanges between working skeletal muscle and heart [8,9], brain [10,11], liver and kidneys [[12], [13], [14]], astrocytes and neurons [15] and *viceversa* (i.e., neurons and astrocytes) [16].

The physiological range of tissue [lactate] is 0.5–20 mM; in muscles and arterial blood of resting healthy humans its concentration is approximates to 1.0 mM and increase more than an order of magnitude under exercise. According to Stegmann et al. [17], it is not possible to use a fixed value for the optimal plasma lactate concentration because it may vary between individuals from 1.4 mM to 7.5 mM. Brooks 2020 [7] reported that repeated lactate exposure from regular exercise results in major effects on the expression of regulatory enzymes of glycolysis and mitochondrial respiration. Besides, it has been recently proposed that lactate is the fulcrum of metabolic regulation *in vivo* [7].

Heart is one of the major recipients of exogenous lactate. The mammalian heart has a very high-energy demand, as it must contract incessantly to guarantee oxygen supply to all organs in the body. For this reason, heart metabolism is highly flexible and can vary widely depending on energy substrate availability. While the majority of ATP production in cardiomyocytes relies on combustion of free fatty acids, glucose and lactate oxidation also significantly contribute to the overall energy homeostasis. In humans during moderate intensity exercise, the contribution of fatty acids fell from 34 to 21 %, while the contribution of lactate increased from 29 to 62 % [18]. In anesthetized dogs, the contribution of lactate to cardiac oxidative energy production increased to 87 % when the arterial lactate concentration exceeded 4.5 mmol/L [19] suggesting that heart depends heavily on exogenous lactate as a fuel when cardiac work is elevated [20,21]. However, the role of lactate as energy substrate and myokine is not fully understood.

It has been demonstrated that regular exercise, which is characterized by regular elevated blood lactate concentrations, enhances cardiac function, increases myocardial mass and stimulates cardiomyogenesis in injured and uninjured adult mouse heart [22].

Attracted by the multiple identities of lactate as energy substrate, metabolite and signal molecule, in this work we investigated the metabolic flexibility of cardiomyocytes analysing their metabolism and morphology in accordance with the carbon source. To deepen the cells response, we growth the AC16 human cardiomyocyte cell line in glucose 5.5 mM (glucose growth, GG) or lactate 8 mM (lactate growth; LG) assessing viability, cell cycle activity, oxidative stress and metabolism. Our data indicated that cardiomyocytes grown on lactate for 72 h showed an increase in cytoskeletal proteins synthesis and hypertrophy in comparison to glucose-grown cells. Interestingly, lactate exposed cells showed an adaptative phase of 48 h characterized by growth arrest and mild cells death that needs further investigations.

## 2 Materials and methods

### 2.1 Cell culture materials and chemicals

AC16 Human Cardiomyocyte Cell Line, sodium l-lactate (L7022) and d-glucose (G7021) were purchased from Merck (Darmstadt, Germany). Dulbecco's Modified Eagle's Medium (DMEM) low glucose (ECM007L) was purchased from Euroclone (Milan, Italy), while DMEM without d-glucose and sodium pyruvate were purchased from Gibco (11,966–025, Thermo Fisher Scientific, Waltham, MA, USA).

### 2.2 Cell culture

AC16 cells were maintained in p100 dishes in DMEM low glucose medium (1 g/L) supplemented with 10 % Fetal Bovine Serum (FBS; Euroclone, Milan, Italy), 100 units/mL penicillin and 100 μg/mL streptomycin at 37 °C and 5 % CO_2_. All the experiments were performed between passage 5–10 and as recommended, the cells were not used beyond the tenth passage [23].

To perform experiments described below, cells were seeded in different multiwell plates keeping the density of 9–10 × 10^3^ cells/cm^2^. When cells reach the 80 % of confluence (after 3 days) media was replaced with DMEM without d-glucose and sodium pyruvate (Gibco, Thermo Fisher Scientific, Waltham, MA, USA) supplemented with 10 % FBS and cells are treated with sodium l-lactate (LG) or d-glucose (GG) at a final concentration of 8 mM and 5.5 mM, respectively. Cells were incubated at 37 °C in a humidified cell culture chamber with 5 % CO_2_ and cell media was replaced every 36 h.

### 2.3 Cell count

AC16 were seed in a 24 well plates and treated as describe above. Three wells per condition, at each time point, were count in double. The counting of cells was performed before and during the entire exposure. Cells were detached with trypsin and resuspend in media. Cells were count using the Burker chamber and optical microscope.

### 2.4 Cytotoxicity assays

AC16 were seed in a 6 well plates and treated as describe above. At each time point (24-48-72 h) cell death was measured determining the extracellular concentration of Lactate Dehydrogenase (LDH) by CytoTox 96® Non-Radioactive Cytotoxicity Assay (Promega, Madison, Wisconsin, USA), following the manufacturer instructions. Absorbance was measured at 490 nm using a Sinergy H1 plate reader (BioTek, Winooski, Vermont, USA).

### 2.5 Cell cycle assay

AC16 were seed in p60 dishes and treated as describe above. After 72 h of growth cells were trypsinized, washed with PBS, resuspended in 0.5 mL of PBS. BD Cycletest Plus DNA Reagent Kit (BD Bioscience, Becton Dickinson Europe Holdings SAS - Francia) was used for cell cycle analysis. Cell cycle distributions were measured using BD Fluorescence-activated cell sorting (FACS) Canto II (BD Biosciences, Becton Dickinson Europe Holdings SAS - Francia).

### 2.6 Measurement of intracellular reactive oxygen species (ROS)

The level of intracellular ROS was assessed by measuring the oxidation of the probe dichlorofluorescin diacetate (DCFH-DA) 10 μM in Dimethyl sulfoxide (DMSO) according to the method of Galli et al., 2018 [24], with slide modifications. Briefly, cells were treated for 72 h in a 24 well plate as reported above and then the medium was replaced with Phosphate Buffered Saline (PBS) containing the probe.

Fluorescence was measured every 10 min with a Sinergy H1 plate reader (BioTek Winooski, Vermont, USA) at excitation/emission wavelengths of 485/538 nm, for a total of 40 min.

After incubation, cells were lysed with RIPA buffer (150 mM NaCl, 100 mM NaF, 2 mM EGTA, 50 mM Tris HCl pH 7.5, 5 mM orthovanadate, 1 % Triton, 0.1 % Sodium dodecyl sulphate (SDS), and 0.1 % protease inhibitor cocktail), centrifuged at 10,000×*g* for 10 min, and total protein content was determined by Bradford assay. Values were expressed as percentage of fluorescence intensity normalized by total protein content.

### 2.7 S-trap digestion of proteins

For the proteomics analysis, cellular pellets were lysed by using denaturing buffer (6 M urea, 1.5 M thiourea, 3.5 mM SDS, 2 mM Ethylenediaminetetraacetic acid (EDTA), 10 mM Dithiothreitol (DTT), 20 mM Tris/HCl) supplemented with the protease Inhibitor Cocktail (Merck KGaA, Darmstadt, Germany) and by repeated cycles of freezing, thawing and sonication. The lysates were then subjected to a classical protocol of protein precipitation by adding four volumes of cold acetone. The samples were centrifuged for 10 min at 10,000 *g* and the pellets resuspended in denaturing buffer (SDS 5 % in AMBIC 50 mM) in agreement with the instructions of S-Trap™ mini spin column digestion protocol (Protifi, Fairport, NY 14450, United States). The cysteines were reduced with 20 mM dithiothreitol and incubated at 95 °C for 10 min. After cooling the samples, the reduced cysteines were alkylated with 40 mM iodoacetamide and incubated in the dark for 30 min at room temperature. The alkylation was then stopped by acidifying the solution with 1 % phosphoric acid. Particulate of colloidal proteins was formed by adding 150 μL of S-Trap binding buffer (90 % aqueous methanol, 100 mM AMBIC, pH 7.1) to the protein solution. The mixture was placed on micro S-Trap columns and centrifuged at 4000 *g* for 30 s. The columns were washed three times with 200 μL of S-Trap binding buffer and discarded after each wash step and centrifuged at 4000 *g* for 30. Each sample was subjected to enzymatic digestion by using a trypsin (Sigma, Milan, Italy) solution (0.12 μg/μL) at an enzyme-to-substrate ratio of 1:20 (w/w). The digestion was performed for 1 h at 47 °C. Peptides were eluted with 80 μL of 50 mM AMBIC followed by 80 μL of 0.2 % aqueous formic acid, 40 μL of 50 % acetonitrile and finally 40 μL of 70 % acetonitrile both containing 0.2 % HCOOH. The peptides were dried under vacuum and finally resuspended in 50 μL of 0.1 % HCOOH for a further LC-MS/MS analysis.

### 2.8 LC-MS/MS analysis

A 6520 Accurate-Mass Q-TOF LC/MS system (Agilent Technologies, Santa Clara, CA, USA) equipped with a 1200 HPLC system and a chip cube (Agilent Technologies, Santa Clara, CA, USA) was used to analyse the peptide mixtures. 1 μL of the peptide mixture was injected by an autosampler and desalted at a flow rate of 4 μL/min in a 40 nL enrichment column with 0.1 % HCOOH as eluent. A C18 reverse-phase capillary column (75 mm × 43 mm, 100A) included into an Agilent Technologies chip (Santa Clara, CA, USA) was used to fractionate the sample at a flow rate of 400 nL/min, with a linear gradient of eluent B (0.1 % HCOOH in 95 % ACN) in A (0.1 % HCOOH in 2 % ACN) from 5 to 80 % in 50 min.

Data-dependent acquisition of one MS scan in the mass range *m*/*z* 300–2400 was carried out, followed by an MS/MS scan of the five most abundant ions in each MS scan. MS/MS spectra were measured automatically when the MS signal was greater than the threshold of 5000 counts. Charge ions preferably isolated were double, triple, and quadruple, and they were fragmented over singly charged ions.

#### 2.8.1 Proteomics data analysis

Raw data files were processed by using MaxQuant software (1.6.8.0 version) [25]. The following parameters were used for raw data processing: trypsin enzyme speciﬁcity, 3 missed tryptic cleavages, oxidation of methionine, formation of pyroGlu from N-terminal glutamine (Q) or glutamic acid (E), β-hydroxybutyryl-lysine and β-succinyl lysine as variable modiﬁcations and cysteine (C) carbamidomethylation as a ﬁxed modiﬁcation. Identification parameters included minimum peptide length of 5 amino acids, minimum of 1 peptide (both razor and unique peptide). Peptide tolerance of 10 ppm, fragment mass tolerance of ±0.02 Da. All proteins were ﬁltered according to a false discovery rate (FDR) of 0.01 % applied both at peptide and protein levels and a maximum peptide posterior error probability (PEP) of 1. The derived peak list generated by Quant. exe (the ﬁrst part of MaxQuant) was searched using the Andromeda search engine integrated into the MaxQuant against the specific fasta file of Homo sapiens obtained from the UNIPROT web site.

#### 2.8.2 Data visualization

MaxQuant output files were subsequently processed using Perseus (version 1.6.8.0) [26] software platforms. An experimental design template was used to get merged replicate experiments (each data set contained two technical replicates) into a single column containing all the proteins into every sample. Contaminants, reverse, and only identified by site hits were filtered out. Expression values of LFQ intensity were log2 transformed and only the protein rows containing a minimum of 2 valid values were maintained within Perseus matrix. Missing values were replaced by random numbers drawn from a normal distribution with a width of 0.3 and a down shift of 1.8. The complete list of proteins (332-dataset) included UniProt ID, gene name, protein name, score, and sequence coverage % (Supplementary Material Table SX). Finally, the proteins were grouped for KBs/C LFQ intensity ratio and ordered for upregulated (>1.2-fold change) and downregulated (<0.8-fold change).

For some peptides, profiling maps of the amino acid sequence were visualized using the Peptigram web application (http://bioware.ucd.ie/peptigram/). Relative amounts of peptides were inferred from the MS signal ion count. In resulting graphs, peptide entries and their corresponding abundances represented with different green shades were aligned to the parent protein sequence.

The MaxQuant file (protein.txt) was further uploaded on Perseus software [26] to perform the statistical analysis. Contaminants, reverse, and only identified by site hits were filtered out. Expression values of LFQ intensity were log2 transformed and only the protein rows containing a minimum of 2 valid values were maintained within the final matrix. PCA analysis was performed by using Perseus Finally, multiple significance test was performed by Perseus to obtain the dataset of significant proteins.

#### 2.8.3 String and KEGG pathways software

The STRING software was used to integrate all known and predicted associations between upregulated proteins, including both physical interactions as well as functional associations [27].

### 2.9 Morphological studies

For morphological optical microscopy evaluation, AC16 cells were seeded in 6 well plates and pictures were taken using a Nikon Eclipse TS100 inverted microscope equipped with a DS-Fi1 camera (Japan).

For Cell morphology assessment by fluorescence microscopy, 72 h treated cells were stained by DAPI/phalloidin as reported by Nolfi et al., 2020, [28]. DAPI (4′,6-diamidino-2-phenylindole dihydrochloride) (1 μg/mL; Cat# D9542) and phalloidin TRITC (phalloidin-tetramethylrhodamine B isothiocyanate) (1 μg/mL; Cat# P1951) (Sigma, Milan, Italy) staining were used for the cell nucleus and the cell actin-cytoskeleton, respectively, and images were captured using a confocal fluorescence microscope Leica TCS SP8.

### 2.10 Western blot analysis

Cells were lysed for 10 min on ice in 80 μl of RIPA buffer. Clarified lysates were obtained by centrifugation at 14,000 *g* at 4 °C for 10 min. The total protein content of each sample was obtained using the Bradford assay (Bio- Rad Laboratories, Hercules, USA). 30 μg of total proteins for each sample were separated by SDS- PAGE and transferred onto Polyvinylidene fluoride (PVDF) membranes. PVDF membranes were incubated with the target primary antibody (*anti*-PDH-E1α; Santa Cruz Biotechnology; Cat# sc-377,092) 1:1000 in 2 % non-fat dry milk solution (PBS, 0.05 % tween) at 4 °C for 24 h and with horseradish-conjugated secondary antibody 1:5000 for 1 h at room temperature. PVDF membranes were washed 3 times in washing solution (PBS, 0.1 % tween) for 10 min and then a chemiluminescence reaction was achieved by probing PVDF membranes with electrochemiluminescence (ECL; Bio- Rad Laboratories, Hercules, USA; Cat#1705060S). Target protein bands have been detected and analysed using Amersham Imager 600 and ImageJ software, respectively. The expression level of the target protein was obtained using Coomassie-stained PVDF membranes for normalization.

### 2.11 Oxygen consumption rate (OCR) analysis

After 72 h of exposure, cells were detached and suspended in 1 mL of medium. The cell suspension was transferred to an airtight thermostatic chamber maintained at 37 °C. Cardiomyocytes oxygen consumption, measured by using a Clark-type O_2_ electrode (Oxygraph Hansatech) for 10 min, has been achieved by taking the rate of oxygen consumption (nmol/min/ml) as an index of respiratory ability. The value of oxygen consumption rate was normalized on the total protein content of each sample.

### 2.12 Metabolomics analysis by GC-MS

Gas chromatography-mass spectrometry (GC–MS) analysis of cardiomyocytes intracellular metabolomic profile was performed using selected ion monitoring (SIM) mode MS, as reported by Pinto et al., 2022 [3], with slide modifications. Briefly, 72 h glucose or lactate treated cardiomyocytes were scraped in 400 μL of 80 % methanol containing 1 μg/mL norvaline (Cat #53721) as internal standard and phase separation was achieved by centrifugation at 4 °C. The methanol-water phase containing polar metabolites was separated and dried using a vacuum concentrator. Dried polar metabolites were dissolved in 10 μl of 40 mg/mL methoxamine hydrochloride in pyridine (Pierce, ThermoFisher Scientific) and kept at 37 °C for 90 min. After dissolution and reaction, 50 μl of MBTSTFA (Cat #375934) was added to the samples that were then incubated at 60 °C for 30 min. Data acquisition was performed by Intuvo 9000 GC/5977B MS System (Agilent Technologies, Santa Clara, CA, USA) equipped with an HP-5MS capillary column (30 m × 0.25 mm x 0.25 μm). 1 μL of each sample was injected in split or split less mode using an inlet liner temperature of 240 °C. GC runs were performed with helium as carrier gas at 1 mL/min. The GC oven temperature ramp was from 70 °C to 280 °C. The temperature of 70 °C was held for 2min. Then, the first temperature ramp was from 70 °C to 140 °C at 3 °C/min. The second ramp was from 140 °C to 150 °C at 1 °C/min. The third temperature ramp was from 150 °C to 280 °C at 3 °C/min. The measurement of metabolites was performed under electron impact ionization at 70 eV using a SIM mode. The ion source and transfer line temperature were set to 250 °C and 290 °C, respectively.

For data analysis, the MS Quantitative Analysis software (Agilent) was used and for determination of relative metabolite abundances, the integrated signal of selected ions for each metabolite was normalized by the signal of the norvaline and by protein content.

### 2.13 Glucose uptake

Cells were plated in a 6 well plate and treated as reported above. After 72 h of incubation with glucose or lactate medium, 40 μM of 2-NBDG (Cat# N13195, Invitrogen, Thermo Fisher Scientific, Waltham, MA, USA) was added in each well. After 2 h, medium was removed, and cells detached using trypsin. Collected cells were washed with PBS and then analysed using a FACS Canto II flow cytometer (BD Bioscence, Becton Dickinson Europe Holdings SAS - Francia). All tests were carried out in triplicate.

### 2.14 Statistical analysis

Data are presented as means ± standard deviation (SD) from at least three experiments and statistical analysis were performed by unpaired Student's T-test using GraphPad Prism 6.01. p < 0.05 were considered statistically significant (*p < 0.05; **p < 0.01; ***p < 0.001; ****p < 0.0001).

## 3 Results

### 3.1 Effect of lactate on cardiomyocyte growth, survival and redox state

Since the major goal of this study was to evaluate the cardiomyocytes metabolic flexibility, we used media contain glucose or lactate as the main carbon sources. The concentrations of both compounds were chosen in the physiological range found in plasma, 5.5 mM for glucose and 8 mM for lactate [29,30]. As preliminary experiment, we evaluated the cell duplication rate counting them at 0-24-48 and 72 h of incubation with glucose (GG) or lactate (LG).

As reported in Fig. 1 panel A, the GG cells start to increase immediately and growth for all the duration of the experiment. On the contrary LG cells showed an initial reduction in growth up to 48 h in comparison to GG cells (p < 0.001), and then start to growth with a comparable duplication rate of GG, as suggested by the similar angular coefficient that describe the pendency of the 48–72 h lines in both the experimental conditions (GG = 2708 vs LG = 3111). At each analysed time point of the curve, GG had a great cell number than LG (p < 0.001). However, from 48 to 72 h both GG and LG cells showed the same growth rate.

**Fig. 1:**
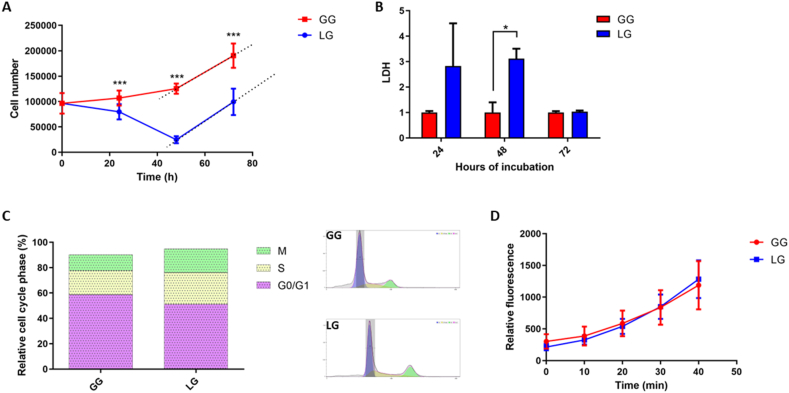
Effect of carbon source on cardiomyocyte growth, survival, and redox state. **A**) Growth curves of AC16 cardiomyocyte cell line in medium supplemented with glucose 5.5 mM (red, GG) or with lactate 8 mM (blue, LG). **B**) Lactate Dehydrogenase (LDH) levels in GG (red) or LG (blue) after 24, 48 and 72 h of growth. **C**) Cytofluorimetric analysis of the cell cycle distribution phases in AC16 cardiomyocyte growth for 72 h with glucose 5.5 mM (GG) or lactate 8 mM (LG). M = mitotic phase cells, S = synthesis phase cells, G0/G1 = cells in G0 or G1 phases. **D**) Measurement of ROS levels in AC16 cardiomyocyte cell growth for 72 h with glucose 5.5 mM (red, GG) or lactate 8 mM (blue, LG) by using the 2′-7′-Dichlorodihydrofluorescein diacetate assay (DCFH-DA) (*p < 0.05; ***p < 0.001). (For interpretation of the references to colour in this figure legend, the reader is referred to the Web version of this article.)

We evaluated extracellular concentration of LDH as marker of cell death released into the cell culture medium when plasma membrane is damaged [31,32]. The results, reported in the histogram of Fig. 1 panel B, confirmed that at the beginning LG cardiomyocytes were subjected to an adaptation phase characterized by cell death. In fact at 24 h, although not statistically significant (p = 0.131), the LDH levels increases. At 48 h the differences become significant (p = 0.002) showing a 3-fold increase. Interestingly, at 72 h, the extracellular concentration of LDH showed similar value in LG and GG (p = 0.472) confirming the overcoming of the adaptation phase.

To verify the similar growth rate at 72 h in LG and GG, we performed a cell cycle analysis using flow cytometry, and the results are reported in Fig. 1 panel C. The distribution of cell cycle phases in LG compared to GG cells reveals similar distribution in the G0/G1, S, and M phases indicating that lactate exposure do not cause significant cell cycle shift among G0/G1 to S and G2/M phases after 72 h of growth.

According to Brooks et al., 2020 [7], disturbances in the normal redox state of cells can be caused by toxic effects of lactate through the production of peroxides and free radicals (ROS) that damage all components of the cell. These authors declare that lactate increase the production of mitochondrial ROS through the activity of the electron transport chain. Furthermore, the second pathway by which mitochondrial lactate metabolism can generate ROS is through a flavin-dependent lactate oxidase localized in the mitochondrial intermembrane space [7,33]. Therefore, we evaluated the redox state in cardiomyocytes at 72 h by the 2′-7′-Dichlorodihydrofluorescein diacetate assay (DCFH-DA), one of the most widely used probe to measure the reactive oxygen species.

We incubated the LG and GG cells for 40 min with the probe, monitoring the fluorescence every 10 min. The results reported in Fig. 1 panel D showed no significant differences between the two curves highlighting that the exposure with 8 mM lactate for 72 h does not cause changes in the redox state in comparison to glucose exposure.

In conclusion, these results suggested that cardiomyocytes growth with lactate undergo to an adaptation phase for 48 h characterized by growth arrest and then they re-start to growth in a similar manner to GG cells, without relevant oxidative stress. Therefore, we decided to perform all the other experiments at 72 h to characterize the features of cardiomyocytes adapted to growth with lactate as carbon source.

### 3.2 Proteomic analysis of lactate treated cardiomyocytes

In order to understand how lactate growing affected protein abundance in cardiomyocytes we analysed proteomic profile of LG and GG cells after 72 h of incubation using LC-MS/MS technique.

The raw data were processed by using MaxQuant software to identify and differently quantify the proteins expressed in LG versus GG sample by label free quantification approach. A total of 265 proteins was identified by using stringent criteria setting a value of 0.01 FDR at the peptide spectrum match level, corresponding to a 99 % confidence score. MaxQuant output files were subsequently processed using Perseus software by filtering the proteins according to criteria reported in material and methods to get a dataset of 74 proteins (roughly 30 % of proteins). Among them, 38 proteins resulted to be up-regulated while 36 were down-regulated. The dysregulation was evaluated in terms of fold change expressed as the ratio of LFQ intensity of 8 mM lactate versus 5.5 mM glucose. The proteins showing a fold change higher than 1.3 or lower than 0.8 were deregulated while the others did not display any change. The PCA analysis of replicates performed by using Perseus reported a strong clustering between the three biological replicates of 8 mM lactate and 5.5 mM glucose (Fig. S1), suggesting a good reproducibility of data.

**Fig. 2:**
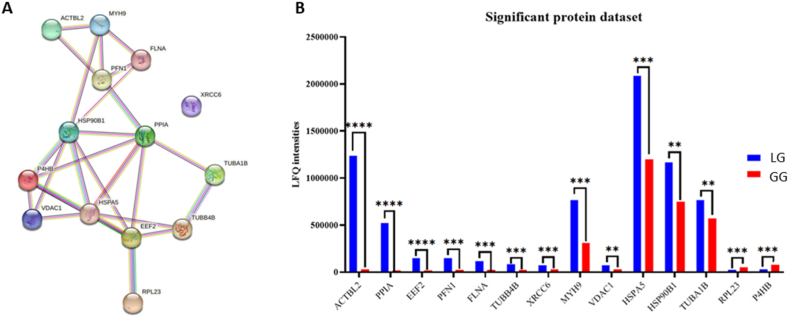
LC-MS analysis of 72-h growth cells in presence of glucose (GG) or lactate (LG) as carbon source; **A)** STRING representation of 14 statistically relevant proteins. **B)** Dysregulation of 14 statistically relevant proteins LG (A-blue bar) versus GG (B-red bar). (For interpretation of the references to colour in this figure legend, the reader is referred to the Web version of this article.)

Within the 74-dataset, further filtering was performed to chase those proteins resulted to be statistically significant (p < 0.05) to a number of 14 proteins (Table 1). Among these proteins, 12 resulted to be upregulated and only two downregulated (C9JD32, p = 0.006; A0A7P0TA71, p = 0.002) in LG versus the GG samples. The subset of 15 proteins was analysed by STRING to highlight possible interaction pathways (Fig. 2 panel A). LFQ intensities for 14-protein dataset were also reported in a histogram representation with the calculated p-value (Fig. 2 panel B).

**Table 1:** List of significantly changed proteins identified in AC16 cardiomyocytes by Liquid Chromatography–Mass Spectrometry (GC-MS) analysis.

Protein name	ID Uniprot	Mol. weight [kDa]	Fold change	Dysregulation	p-value	Peptides	Score	Sequence coverage [%]
Beta-actin-like protein 2 OS	Q562R1	42.003	40.262	Up-regulated	<0.001	6.000	13.784	25.800
Peptidyl-prolyl *cis*-trans isomerase A OS	P62937	18.012	25.374	Up-regulated	<0.001	7.000	51.898	64.200
Elongation factor 2 OS	P13639	95.337	6.961	Up-regulated	<0.001	8.000	71.272	15.700
Profilin-1 OS	P07737	15.054	6.580	Up-regulated	0.001	3.000	43.105	22.900
Filamin-A OS	Q60FE5	278.220	5.127	Up-regulated	0.003	10.000	68.902	7.700
Tubulin beta-4B chain OS	P68371	49.830	3.286	Up-regulated	0.003	16.000	47.601	50.600
ATP-dependent DNA helicase 2 subunit 1 OS	B1AHC9	64.283	2.575	Up-regulated	0.008	5.000	53.669	12.500
Myosin-9 OS	P35579	226.530	2.443	Up-regulated	0.001	41.000	306.270	28.200
Voltage-dependent anion-selective channel protein 1 OS	P21796	30.772	2.403	Up-regulated	0.016	4.000	27.431	19.400
Endoplasmic reticulum chaperone BiP OS	P11021	72.332	1.742	Up-regulated	0.002	36.000	309.950	55.000
Endoplasmin OS	A0A087WT78	86.589	1.554	Up-regulated	0.017	18.000	164.450	27.600
Tubulin alpha-1B chain	P68363	50.151	1.335	Up-regulated	0.015	16.000	151.420	44.300
60S ribosomal protein L23	C9JD32	9.669	0.473	Down-regulated	0.006	2.000	14.488	20.900
Protein disulfide-isomerase	A0A7P0TA71	62.031	0.370	Down-regulated	0.002	5.000	38.374	18.900

Seven proteins such as actin, myosin, filatin-A, endoplasmin, tubulin α and tubulin β were involved in cytoskeleton organization. This finding resulted to suggest a reorganization in contractile cytoskeleton activity in LG cells. As demonstrated by Karbassi et al., 2020 [34], dynamic changes structurally and functionally of cardiomyocytes took place during *in vivo* maturation in combination with an increase in cell size and a remodelling of nuclei, junctions and other organelles. Other authors observed cytoskeletal changes in hearts of patients affected from chronic kidney as a result of metabolic abnormalities [35].

Moreover, other proteins Peptidyl-prolyl *cis*-trans isomerase A (P62937) and Endoplasmic reticulum chaperone BiP OS (P11021) involved in the unfolded protein response and protein quality control occurring in endoplasmic reticulum, were upregulated. This finding was confirmed by many authors who demonstrated that external stimulations and alteration of the metabolic environment of cardiomyocytes activated stress-response pathways which can act as key regulator of cell function, growth and survival [[36], [37], [38], [39]].

### 3.3 Chronic lactate exposure induces cytoskeleton modification on cardiomyocytes

Our proteomic analysis identified the overexpression of several cytoskeleton proteins in LG cells. Moreover, data from literature demonstrated that lactate is able to influence cell cytoskeletal architecture, promoting cell morpho-functional alterations [40].

**Fig. 3:**
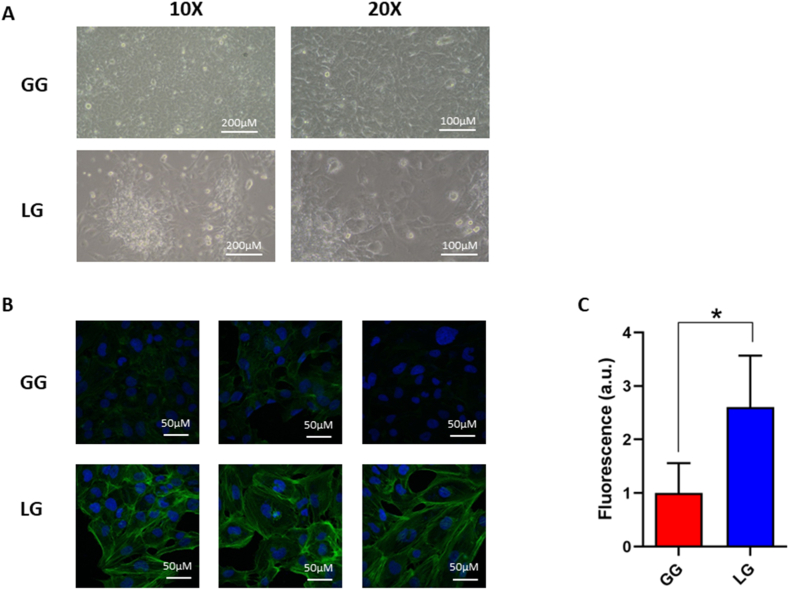
Effect of lactate on cardiomyocyte morphology and cytoskeleton. **A**) Representative cell morphology images, by optical microscopy, of AC16 cardiomyocyte growth in glucose 5.5 mM (GG) or lactate 8 mM (LG) for 72 h. **B**) Representative confocal microscopy images of microfilament pattern by a phalloidin/DAPI staining in AC16 cardiomyocyte in GG and LG cells at 72 h. **C**) Quantification of relative phalloidin fluorescence performed by ImageJ software. Histogram reports the mean values and standard deviations of four image from three independent biological experiments (*p < 0.05).

Analysing the cells morphology by optical microscopy, we observed the presence of two morphologically distinguishable cell populations between the LG and GG cells. It is interesting to note that at 72 h LG cells formed large cell aggregates in comparison to non-treated cells as reported in Fig. 3 panel A.

In the light of these observations, we evaluated the possible effects of 72 h of lactate exposure on microfilament organization. Herein, we report microfilament distribution pattern by a phalloidin/DAPI staining, which is commonly used to stain actin filaments in cells and tissues and observing cells by confocal microscopy [41]. We reported the results in Fig. 3 panel B. Quantification of relative fluorescence was performed by ImageJ software and the relative fluorescence intensity of LG cells is increased by 2.6 fold (p = 0.0281) in comparison to GG cells suggesting a general increase in cytoskeleton proteins expression level (Fig. 3 panel C).

### 3.4 Chronic lactate exposure affects cardiomyocytes metabolism

**Fig. 4:**
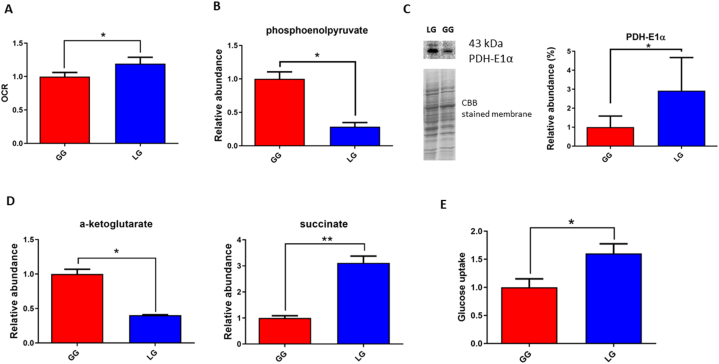
Lactate exposure affects cardiomyocytes metabolism. **A**) OCR measurement in AC16 cardiomyocyte growth with glucose 5.5 mM (red bar, GG) or lactate 8 mM (blue bar, LG) for 72 h. **B**) Relative abundance of phosphoenolpyruvate by metabolomic GC-MS analysis reported as fold decrease in LG (blue bar) respect to GG (red bar) cells. **C**) Relative abundance of alpha-ketoglutarate and succinate. **D**) Representative immunoblot image and histogram of PDH-1Eα; normalization of immunoblot was performed on Coomassie-stained PVDF membrane (original image of Western blot is reported in Fig. S2). **E**) Glucose uptake in AC16 cardiomyocyte growth in glucose 5.5 mM (GG) or lactate 8 mM (LG) for 72 h. All histograms display the results as mean values and SD of three independent biological experiments (*p < 0.05; **p < 0.01). (For interpretation of the references to colour in this figure legend, the reader is referred to the Web version of this article.)

To analyse metabolic changes induced by continuous lactate availability as main carbon source (72 h) we first evaluated the oxygen consumption rate in both LG and GG cells. The metabolic activity of cells using lactate as a carbon source, instead of glucose is mainly oxidative so we expect an increase in the rate of oxygen utilization by cells treated with 8 mM lactate. Indeed Hui et al., 2017 [42], reported that glucose feeds the TCA cycle via circulating lactate, implicating lactate as an important energy source for the myocardium. These authors demonstrated that, when lactate is added to the medium, it rapidly increased the cellular oxygen consumption rate. From this perspective we determined the oxygen consumption rate as reported in Materials and Methods. The results shown in Fig. 4 panel A, highlight a significant increase of 20 % in OCR (p = 0.0438) by LG cells and we confirm what reported by Brooks 2018 [43] that heart lactate disposal is by mitochondrial respiration.

In order to confirm these observations and to highlight which molecules are modified under our experimental conditions, we performed intracellular metabolomic analysis, focusing on Krebs cycle metabolites, amino acids and glycolysis. In Table 2 is shown the metabolic profile of LG cells compared with the GG, through GC‐MS as reported in Materials and Methods.

**Table 2:** Metabolic profile of AC16 cardiomyocytes by Gas Chromatography–mass spectrometry (GC-MS) analysis.

Metabolite name	CAS number^◘^	KEGG ID°	Fold change^+^	p- value^Δ^
l-alanine	56-41-7	C00041	1,27	0,1238
l-asparagine	70-47-3	C00152	1,79	0,0294*
l-aspartic acid	56-84-8	C00049	4,12	0,0089*
l-phenylalanine	63-91-2	C00079	1,92	0,0248*
l-glycine	56-40-6	C00037	1,88	0,0261*
l-glutamic acid	56-86-0	C00025	2,03	0,0249*
l-glutamine	56-85-9	C00064	1,64	0,0546
l-isoleucine	73-32-5	C00407	2,03	0,0213*
l-histidine	26,062-48-6	C00135	1,75	0,0480*
l-leucine	61-90-5	C00123	2,10	0,0184*
l-methionine	63-68-3	C00073	2,14	0,0167*
l-proline	147-85-3	C00148	0,83	0,3803
l-serine	56-45-1	C00065	2,15	0,0203*
l-tyrosine	200-460-4	C00082	2,06	0,0237*
l-valine	72-18-4	C00183	1,99	0,0225*
Alpha-ketoglutaric acid	328-50-7	C00026	0,40	0,015*
Citric acid	5949-29-1	C00158	0,95	0,6756
Fumarate	110-17-8	C00122	1,24	0,254
Malate	149-61-1	C00149	1,29	0,1471
Succinate	56-14-4	C00042	3,11	0,005*
Phosphoenolpyruvate	138-08-9	C00074	0,28	0,0067*
Pyruvate	57-60-3	C00022	0,95	0,6863
l-lactic acid	79-33-4	C00186	1,57	0,0462*
Palmitic acid	64,519-82-0	C00249	6,30	0,1057
Stearic acid	57-11-4	C01530	3,83	0,0145

^**◘**^ Chemical Abstract Service number.

° KEGG identifier (https://www.genome.jp/kegg/).

+ Ratio between lactate and glucose treated cells.

^**Δ**^ p-value was determined by T-test.

**Fig. 5:**
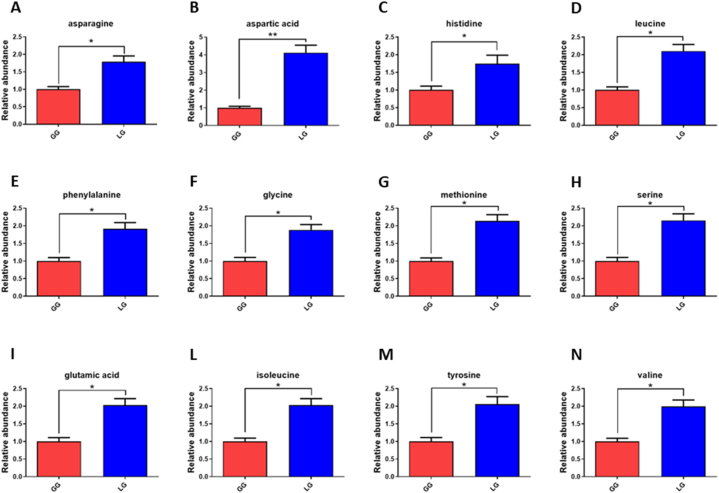
Relative abundance of amino acids by metabolomic GC-MS analysis reported as fold increase (or decrease) in cells growth with lactate 8 mM (blue bar, LG) respect to those treated with glucose 5.5 mM (red bar, GG). **A)** asparagine; **B)** aspartic acid; **C)** histidine; **D)** leucine; **E)** phenylalanine; **F)** glycine; **G)** methionine; **H)** serine; **I)** glutamic acid; **L)** isoleucine; **M)** tyrosine; **N)** valine. All histograms display the results as mean values and SD of three independent biological experiments (*p < 0.05; **p < 0.01). (For interpretation of the references to colour in this figure legend, the reader is referred to the Web version of this article.)

Among the metabolites we observed a decreased level of glycolytic metabolite phosphoenolpyruvate of about 72 % (Fig. 4 panel B; 3.5-fold, p = 0.0067), confirming the reduced rate of glycolysis in LG cells, while we found unchanged the level of pyruvate. As reported in literature [42,44], mitochondrial lactate is converted to pyruvate by lactate dehydrogenase B and addressed into the mitochondrial respiratory tract where it is converted in Acetyl-CoA by Pyruvate dehydrogenase (PDH) that in our LG cells is overexpressed (2.9 fold, p = 0.048; Fig. 4, panel C). As regards the metabolic intermediates of Krebs cycle, we observed a strong increase of succinate (3.1-fold, p = 0.005) and on the contrary a significant decrease of alpha-ketoglutaric acid (2.5-fold decrease, p = 0.015) as reported in Fig. 4 panel D. Since from literature is known that glucose uptake is a feature of increased protein synthesis, cell size and hypertrophy in cardiomyocytes [45], we evaluated glucose uptake in LG and GG cells at 72 h. The results are shown in histogram of Fig. 4 panel E. It is evident that the glucose uptake is increased of about 1.60-fold (p = 0.0349) in LG cells in comparison to GG. Moreover, we found a significant increased levels of several amino acids shown in Fig. 5 such as l-asparagine (1.79-fold, p = 0.0294 – panel A), l-aspartic acid (4.12-fold, p = 0.0089 - panel B), l-histidine (1.75-fold, p = 0.048 – panel C), l-leucine (2.10-fold, p = 0.0184 – panel D), l-phenylalanine (1.92-fold, p = 0.0248 - panel E), l-glycine (1.88-fold, p = 0.0261 - panel F), l-methionine (2.14-fold, p = 0.0167- panel G), l-serine (2.15-fold, p = 0.0203 – panel H), l-glutamic acid (2.03-fold, p = 0.0249 – panel I), l-isoleucine (2.03-fold, p = 0.0213 – panel L), l-tyrosine (2.06-fold, p = 0.0237 – panel M) and l-valine (1.99-fold, p = 0.0225 – panel N). All these data suggest an increase in protein synthesis confirming that cardiomyocytes exposed to lactate as carbon source for 72 h, undergo to an increase in protein synthesis that can lead to the alteration of cytoskeleton and hypertrophy.

## 4 Discussion

The biochemical significance of lactate within the field of myocardial metabolism is under-appreciated, due to an incomplete understanding of its biological functions. We analysed the lactate exposure per se in a cell line model to study how cells respond to this carbon source. In our experimental model, we want to isolate the effects of lactate on cardiomyocytes without the presence of other nutrients to deepen the cellular response to lactate without the interference of other carbon sources. To use a medium containing only lactate can help better understand how cells respond to its accumulation; a medium containing only lactate can be targeted and advantageous to study specific cellular responses related to lactate metabolism. In our experimental model, we want to isolate the effects of lactate on cells without the presence of glucose. This allows the cells' responses to lactate to be studied more precisely.

Lactate is recognized as carbohydrate fuel source and a signalling molecule [43] and it is a regulator of adaptation in skeletal muscle; in a previous paper, we suppose that the ability to tolerate high levels of blood lactate during training period is associated with a success in performance. Besides, Dong et al., 2021 [18], reported that cardiomyocytes have a “metabolic flexibility” and are able to produce ATP from different carbon source such as lactate.

Cellular metabolism and signalling are critical to cardiomyocyte function in adult heart, and we wanted to investigate how a high but non-pathological concentration lactate could induce cytoskeletal remodelling in cultured cardiomyocytes. To deepen the cells response to a high (8 mM) and prolonged (72 h of exposure) concentration of lactate, we investigated the effect of lactate as main carbon source on proteome and metabolome proliferative AC16 cardiomyocytes cell line, since these conditions do not modify growth rate and redox status compared to glucose exposure [46].

In literature, Liu and Proud 2016 [47], reported that exogenous lactate enhances cardiac proliferation and hypertrophy inducing protein synthesis and in our experimental conditions, we found an increase in several amino acids concentration as well as the increase of protein Elongation factor 2 indicating arise in protein synthesis.

According Cluntun et al., 2021 [46], the intracellular accumulation of lactate stimulates lactate dehydrogenase (LDH) towards its oxidation to pyruvate activating pyruvate dehydrogenase (PDH) which converts pyruvate to acetyl-CoA that enters the Krebs cycle.

Lactate, through conversion to pyruvate, can influence the NAD+/NADH ratio within cells. This ratio is crucial for various metabolic reactions, including those involved in fatty acid oxidation. A shift in this ratio may indirectly affect the utilization of fatty acids for energy production [48].

In our experimental model and by Western blot analysis we evidenced an increase in the expression of PDH in LG cells confirming the pyruvate oxidation. Regarding the induction of mitochondrial respiration, we found an increase of oxygen consumption and a reduction of phosphoenolpyruvate (PEP) level; this confirms the increase in mitochondrial respiration that uses the pyruvate produced by exogenous lactate. Our results indicate however that the concentration of pyruvate does not increase and, without modifying the speed of the Krebs cycle, we found arise in the intracellular concentration of succinate. Moreover, the reduced concentration of alpha ketoglutarate observed in LG cells and the contemporary increase of intracellular succinate, confirm that pyruvate is oxidized in TCA cycle. Despite a direct metabolic connection between pyruvate and succinate, pyruvate indirectly influences the production of succinate through its conversion to acetyl-CoA.

In addition, from data reported in literature, accumulation of intracellular succinate is directly responsible for the cytoskeletal modifications and hypertrophy [49,50].

Many authors report that blood levels of succinate increased in numerous pathological conditions and Aguiar et al., 2014 [51] reported for the first time that succinate, through the activation of GPR91, plays an important role in cardiomyocyte hypertrophy.

In our experimental conditions, the increase in intracellular succinate stimulated by the continuous exposure to lactate induces, in cultured cells, a modification of cytoskeleton proteins, remodelling the cell morphology and stimulating the expression of several proteins involved in cytoskeletal composition and associate with myocardial hypertrophy. Indeed, we observed by proteomics analysis the changes in actin level that in cardiomyocytes sarcomere its polymeric F-actin is critical for the contractile function of cardiac muscle [52,53].

Among the various actin-binding proteins (ABPs) associated with cardiac hypertrophy we found as overexpressed the protein profilin-1 that accelerates actin aggregation and increase the development of cardiac hypertrophy. Furthermore Pan et al., 2022 [54] reported that in H9c2 cells the inhibition of profilin-1 can attenuate cardiac hypertrophy.

We found as overexpressed in LG cardiomyocytes several proteins involved in microtubule structure and together with microfilaments, microtubules constitute the major component of the cardiomyocyte cytoskeleton.

Relating to this we found an increase in expression of Tubulin and according to several authors the cardiomyocyte microtubule network is also implicated in hypertrophy, and it is indispensable for cardiac growth via spatiotemporal control of the cardiomyocyte transcription and translation, which promotes the addition of new contractile units [55,56].

Moreover, we found overexpressed Filamin A and Myosin. Filamins are a family of Actin-binding proteins of the cytoskeleton involved in the organization of Actin filaments. In heart muscle, Filamin is found in the sarcolemma, where it interacts with a variety of transmembrane receptors. Regarding Myosin several studies demonstrated that mutations in genes encoding Myosin heavy chain explain hypertrophic cardiomyopathy [57].

Recently it has also observed that cellular distribution of the metabolites aspartate and glutamate is influenced by the modification of the pyruvate-lactate axis. We found in LG cells an increase of both cytoplasmic aspartate and glutamate and Ritterhoff et al., 2020 [58] suggested that an increase in aspartate induces cardiac hypertrophy. The authors identified aspartate accumulation as the key molecule to induce cardiomyocyte hypertrophy, and in addition, Palmieri et al., 2012 [59] found that during hypertrophy, cytosolic aspartate is increased. Mitochondrial pyruvate could regulates mitochondrial aspartate-glutamate antiporter in cardiomyocytes and modulate the localization of both two amino acids resulting in the induction of hypertrophy [58,59].

## 5 Limitation

In this study, to identify the effect of lactate, we compared the results obtained with 8 mM of lactate with those obtained with 5.5 mM glucose and we know that this study design is not close to the real life. Moreover, in the real life, lactate does not stay 72 h at elevated concentrations. As reported in manuscript our idea emerged from the results obtained in our previous studies, where we verified, in plasma from athletes during the training period, a high level concentration of lactate compared to ordinary people of the same age. In addition, from the literature is well known that over the last several decades, lactate is a major energy substrate for skeletal muscle, heart and brain as well as being a signalling molecule. With this in mind, we used one commercial human cardiac cell line to characterize the lactate metabolic profile of myocardium to go into detail to the metabolic flexibility of the heart. The obtained results could help to understand the effect of this metabolite on the heart of high-performance athletes. The biochemical significance of lactate within the field of myocardial metabolism is under-appreciated, due to an incomplete understanding of its biological functions. In our experimental model, we want to isolate the effects of lactate on cells without the presence of other nutrients or substances found in more complex media. This allows the cells' responses to lactate to be studied more precisely. Using a medium containing only lactate can help to better understand how cells respond to lactate accumulation. Despite these limitations, we consider that the findings in this work are very promising and further experiments are needed to clarify whether lactate can induce hypertrophy and remodelling in other cellular models. Continued research in this area will be essential to fully elucidate the complex roles of lactate.

## 6 Conclusion

**Fig. 6:**
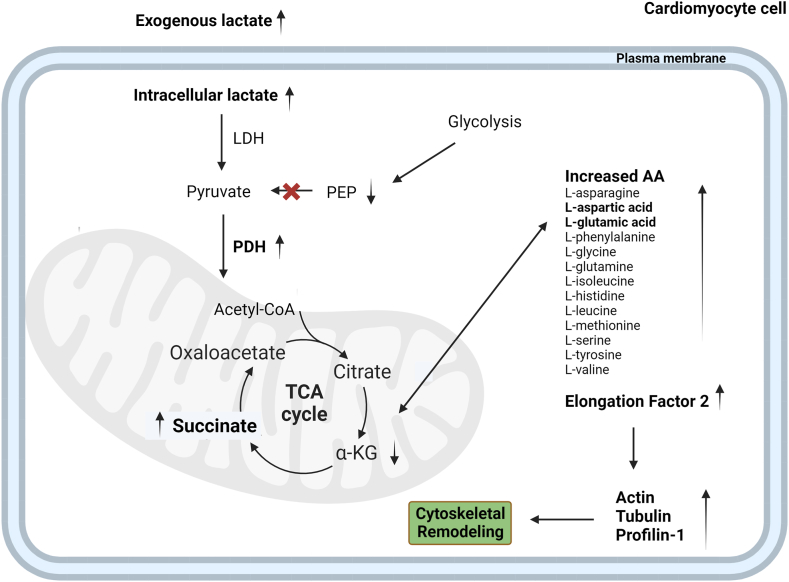
Schematization of endogenous lactate effects on cardiomyocyte cell. Created with BioRender.com.

We can conclude that cultured human cardiomyocytes grew up with lactate as main carbon source, showed both an oxidative metabolism towards mitochondrial respiration and an endocellular succinate production. We proposed that succinate produced is able to stimulate an increase in several amino acids levels that, together with the observed increase in EF2, leads to the stimulation of protein synthesis with the result of an increase in cytoskeletal proteins as reported in the summary Fig. 6. We confirm what some authors reported, therefore upon hypertrophic stimulation, microtubules redistribute new protein synthesis to sites of growth suggesting that properly localized translation is a critical determinant of cardiac hypertrophy.

In addition, the increased level of succinate could be due to the contemporary increase in glutamate that can be converted to succinate and ATP without molecular oxygen. Alpha-ketoglutarate can also participate in an aminotransferase reaction, with aspartate generating oxaloacetate that is metabolized through malate and fumarate to succinate with the generation of ATP [60]. In Fig. 6 we proposed the role of these two amino acids in inducing an intracellular increase in succinate. Interestingly, from a physiologic point of view, among exercise training strategies, high-intensity interval training (HIIT) has been specifically reported to increase blood lactate during and after exercise [61]. High-intensity interval training (HIIT) is a form of interval training that alternates very brief periods (such as 20–40 s) of intense exercise at maximum effort with periods (such as 15–30 s) of less intense exercise [62]. Cellular remodelling induced by high lactate exposure observed in the present study could be the basis for cardiac remodelling induced by HIIT [63]. This is an important perspective because HIIT is now also extended to non-athletes and is increasingly extending to rehabilitation strategies in non-athletes [64].

### Funding

This study was supported by grants from 10.13039/501100004434University of Florence (Grant: 58515_Simonelutiricaten23 and 58515_ALESSANDRAMODESTIRICATEN23)

### Ethics statement

We do not have approval from the ethics committee because the work is done on a commercial cell line (AC16 Human Cardiomyocyte Cell Line from Merk).

## CRediT authorship contribution statement

**Simone Luti:** Writing – original draft, Methodology, Investigation, Data curation, Conceptualization. **Rosamaria Militello:** Investigation, Formal analysis. **Gabriella Pinto:** Validation, Investigation, Formal analysis. **Anna Illiano:** Investigation, Formal analysis. **Riccardo Marzocchini:** Investigation, Formal analysis. **Alice Santi:** Investigation, Formal analysis. **Matteo Becatti:** Investigation, Formal analysis. **Angela Amoresano:** Validation, Investigation. **Tania Gamberi:** Writing – review & editing, Resources, Investigation, Formal analysis. **Alessio Pellegrino:** Resources, Formal analysis. **Alessandra Modesti:** Writing – review & editing, Validation, Supervision, Resources, Funding acquisition, Data curation, Conceptualization. **Pietro Amedeo Modesti:** Writing – review & editing, Supervision.

## Declaration of competing interest

The authors declare that they have no known competing financial interests or personal relationships that could have appeared to influence the work reported in this paper.

## Figures and Tables

**figs1:**
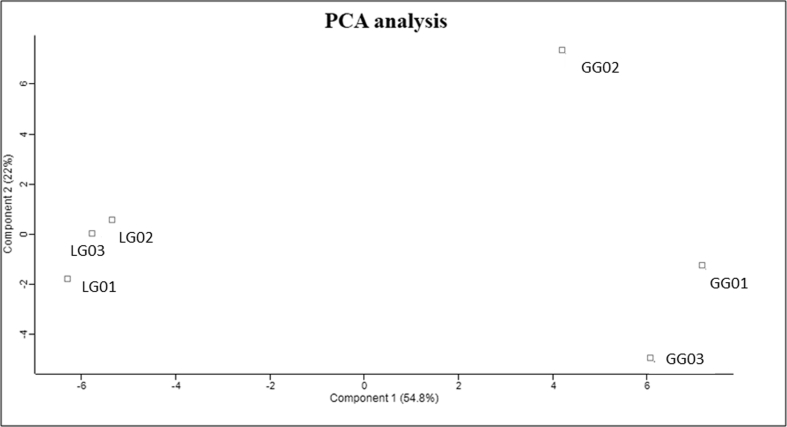


**figs2:**